# Predicting synthetic lethal interactions using conserved patterns in protein interaction networks

**DOI:** 10.1371/journal.pcbi.1006888

**Published:** 2019-04-17

**Authors:** Graeme Benstead-Hume, Xiangrong Chen, Suzanna R. Hopkins, Karen A. Lane, Jessica A. Downs, Frances M. G. Pearl

**Affiliations:** 1 Bioinformatics Lab, School of Life Sciences, University of Sussex, Falmer, Brighton, United Kingdom; 2 Division of Cancer Biology, Institute of Cancer Research, Chester Beatty Laboratories, London, United Kingdom; University of Toronto, CANADA

## Abstract

In response to a need for improved treatments, a number of promising novel targeted cancer therapies are being developed that exploit human synthetic lethal interactions. This is facilitating personalised medicine strategies in cancers where specific tumour suppressors have become inactivated. Mainly due to the constraints of the experimental procedures, relatively few human synthetic lethal interactions have been identified. Here we describe SLant (Synthetic Lethal analysis via Network topology), a computational systems approach to predicting human synthetic lethal interactions that works by identifying and exploiting conserved patterns in protein interaction network topology both within and across species. SLant out-performs previous attempts to classify human SSL interactions and experimental validation of the models predictions suggests it may provide useful guidance for future SSL screenings and ultimately aid targeted cancer therapy development.

## Author summary

Our new algorithm SLant, uses artificial intelligence to help target future cancer drug research. In healthy cells tens of thousands of proteins work together forming large interaction networks. However, in cancerous cells genetic damage means that many of these proteins are disabled. Basic functions like DNA repair and signaling no longer work properly, and the cell replicates without proper control. Recent experience with breast cancer shows that gentler, more personalised therapies can be achieved by finding pairs of proteins which are ‘synthetically lethal’. The term means that the cell can cope if either one of the proteins does not work, but will die if neither of the proteins is functioning. Many synthetic lethal interactions are known, but there are many millions of potential pairs and finding new ones experimentally is difficult and time-consuming. SLant uses most of the experimental data that we have to identify the patterns in the protein interaction network associated with being part of a synthetic lethal interaction. By searching the network for proteins pairs that match these patterns, it can effectively predict new synthetic lethal pairs. The predictions were then checked against the rest of the experimental data. Our predictions are publicly available through the Slorth database.

## Introduction

Despite sustained global efforts to develop effective therapies, cancer is now responsible for more than 15% of the world’s annual deaths. There are over 12 million newly diagnosed cases per annum and this figure continues to grow [1]. Standard chemotherapy involves non-selective, cytotoxic agents that often have limited effectiveness and strong side-effects. Consequently, the current focus in oncology drug discovery has moved towards identifying targeted therapies that promise both improved efficacy and therapeutic selectivity [2].

The development of multi-platform genomic technologies has enabled the identification of many of the genes that drive cancer [3]. These cancer driver genes can be broadly classified either as oncogenes or tumour suppressors. The protein product of an oncogene shows an increase in activity, or a change or gain of function when mutated, whereas mutations or epigenetic silencing in tumor suppressors result in an inactivation or loss of function (LOF) of the protein product [4].

Targeted therapies that act on oncogenes often work by directly inhibiting the activated protein product. This strategy has been particularly successful for targeting nuclear receptor proteins or those that contain protein kinase domains. [5–7]. Unfortunately, it is not usually feasible to repair tumour suppressor genes or their protein products, particularly if they are inactivated by a truncation [8]. Instead an emerging strategy is to target tumour suppressors indirectly by exploiting synthetic lethal interactions.

Synthetic lethality (SSL) is a phenomenon whereby individual genes in a pair can be knocked-out without affecting cell viability, whilst disruptions in both genes concurrently cause cell death [9]. Synthetic sensitive and synthetic sickness interactions are extensions of this concept where concurrent genetic interactions impair cellular fitness without necessarily killing the cell. Conversely, synthetic dosage lethality (SDL) interactions occur when over-expression of one gene, in combination with loss of function in another gene results in cell death. SSL and SDL interactions are both examples of negative genetic interactions. Negative genetic interactions are events where a deviation from the expected phenotype is observed when genetic mutations occur in more than one gene [10].

To exploit SSL interactions therapeutically one gene, the tumour suppressor, is genetically inactivated by mutation while the protein product of the other is targeted and inactivated pharmacologically [11]. Synthetic dosage lethal interactions can be used for targeting cancer cells with over-expressed, undruggable oncogenes [11]. SDL causes cell death as a result of one gene being genetically activated (GOF, the oncogene) and another being inactivated (LOF, the drug target).

PARP inhibitors are the most developed therapies that exploit SSL interactions. The PARP inhibitor Olaparib, has been approved for the treatment of patients with recurrent, platinum-sensitive, high-grade serous ovarian cancer with BRCA1 or BRCA2 mutations [12, 13]. PARP1 (poly(ADP-ribose) polymerase) is an important component in DNA single strand break repair and has been shown to share a synthetic lethal relationship with both BRCA1 and BRCA2 [14, 15], which are themselves both key in DNA double strand break repair. Complete loss of function of the protein product of either BRCA gene leaves cells extremely sensitive to PARP inhibitors presenting this therapeutic opportunity [16, 17].

Other studies have highlighted a range of SSL interactions that may provide suitable targets for therapy [18–20]. For example, PI5P4K kinases are essential in the absence of p53 [21], inhibition of ENO2 inhibits viability in ENO1 deficient glioblastoma cells [22] and APE1 inhibitors in PTEN deficient cells results in the induction of apoptosis [23].

Currently, mainly due to experimental limitations [24] exhaustive experimental identification of human SSL interactions is not tenable. However there are many studies focused on screening for genetic interactions in model organisms [25]. Unfortunately, genetic interactions are not highly conserved between lower eukaryotes and their human orthologue equivalents [26]. Instead, in order to identify novel human SSL interactions, we are left to infer and predict these pairs indirectly from existing human and model organism data through the use of models and other computational techniques [27].

Several classifiers have been developed to predict genetic interactions within model organisms. Wong et al. [28] predicted genetic interactions in *Saccharomyces cerevisiae* using decision tree classifiers with multiple data types and network topology. Paladugu et al. [29] focused on *S*. *cerevisiae* data; by extracting multiple features from protein interaction networks they achieved sensitivity and specificity exceeding 85% using support vector machine (SVM) classifiers. Later, Chipman et al. [30] employed random walks and decision tree classifiers on protein interaction and gene ontology (GO) data to classify both S. *cerevisiae* and *C*. *elegans* negative genetic interactions.

Several classifiers have been developed to predict genetic interactions between species. Zhong and Sternberg [31] classified *Caenorhabditis elegans* negative genetic interactions based on orthologous gene pairs in *S*. *cerevisiae* and *Drosophila melanogaster*. Jacunski et al. [32] developed SINaTRA (Species-INdependent TRAnslation) to classify *S*. *cerevisiae* SSL pairs based on *Schizosaccharomyces pombe* training data and vice versa, using features extracted from physical interaction data. The model trained on *S*. *cerevisiae* data was applied to predict 1,309 human SSL pairs with a reported false positive rate of 0.36. Similarly Wu et al [33] developed MetaSL, an ensemble machine learning mode which applied eight different classifiers on *S*. *cerevisiae* data and applied it to predict human SSL pairs.

Using an alternative approach, the DAISY workflow predicted human SSL interactions directly from human cancer and cell–line data [34]. The authors used somatic copy number variation and mutation profiles to achieve a ROC AUC score of 0.779 demonstrating a strong propensity (p-value < 1e-4) for predicting SSL pairs in *H*. *sapiens*.

There are a number of additional recent studies that use biological networks to predict genetic interactions. Mashup [35] reported an average area under the precision curve (AUPR) of 0.59 for SSL and 0.51 for SDL pair prediction in a real human dataset. Others have utilised gene ontology terms to predict SSLs. These include Ontotype [36], where the authors predict the growth outcome on double knock-out of gene pairs. Their prediction set of gene pairs related to DNA repair and nuclear lumen correlated with Costanzo et al’s [37] validated SSL dataset with a coefficient of r = 0.61. The authors of DCell [38] constructed a visible neural network embedded in the hierarchical structure of 2526 subsystems describing the eukaryotic cell and used this to predict negative genetic interactions in *S*. *cerevisiae*.

In this study we introduce SLant (Synthetic Lethal analysis via Network topology), a random forest classifier trained on features extracted from the protein-protein interaction (PPI) networks of five species. These features comprise both node-wise distance and pairwise topological PPI network parameters and gene ontology data. Using SLant we provide in-species, cross-species and consensus classification for synthetic lethal pairs in all five organisms including human. We subsequently experimentally validated three of the predicted human SSLs in a human cell-line. Finally we identify a large cohort of candidate human synthetic lethal pairs which are available with the consensus predictions for all the model organisms in the Slorth database (http://slorth.biochem.sussex.ac.uk).

## Results

A genome-wide protein-protein interaction (PPI) network was constructed for *Homo sapiens* and each of our model organisms (*S*. *cerevisiae*, *D*. *melanogaster*, *C*. *elegans*, *and S*. *pombe)* using PPI data from the STRING database [39]. In this network, each node represents a protein and each edge represents a physical interaction between two proteins. For each pair of proteins 12 node-wise and 7 pairwise features were extracted from the PPI network using the R igraph library [40]. Each protein in the network was labelled with its respective Ensembl gene identifier so that this physical interaction data could be matched with gene interaction data. For each gene pair 3 additional GO term related features were generated using Gene ontology (GO) data [41].

**Fig 1:**
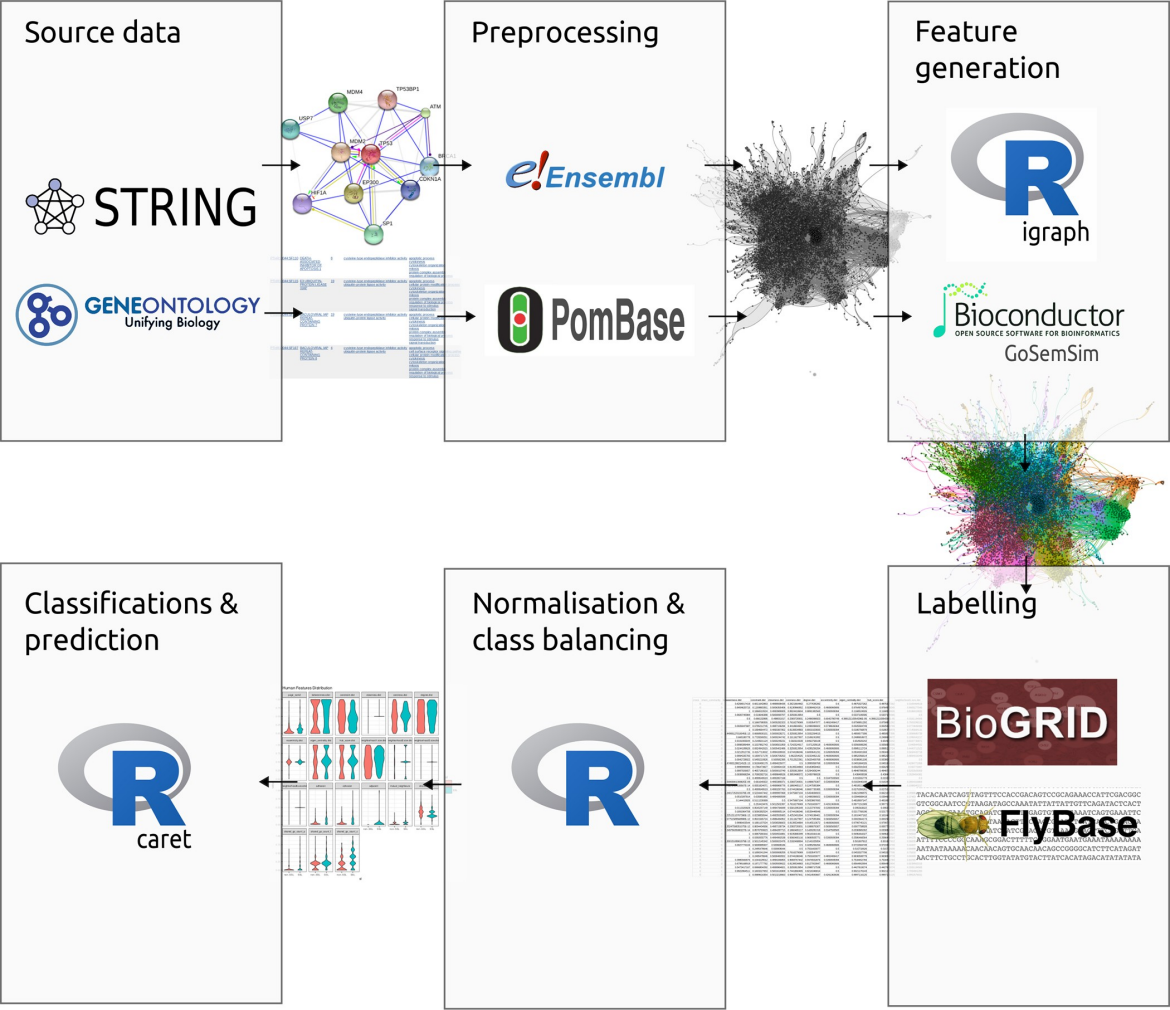
A schematic visualising how SLant’s source data is collated from STRING and the Gene Ontology Consortium, preprocessed so that this source data can be directed joined with BioGRID data for labeling and processed to create the final training set. Feature generation was completed using R, the R igraph library and GoSemSim, a Bioconductor package.

For each PPI network, pairs of proteins whose respective genes were identified as having a negative genetic interaction in BioGRID [42] were labelled as having an SSL interaction (Fig 1). Equal numbers of SSL and non-SSL gene pairs were selected independently for the training sets for each species (see methods). Similarly we created training sets for SDL and non-SDL gene pairs in *H*. *sapiens* and *S*. *cerevisiae*, the only two species where there is enough data for prediction purposes.

### Network parameter distributions in humans

The features used for classification in the SLant algorithm were broadly divided into node-wise, pairwise or GO-term related categories. Node-wise features were derived from an individual node's network parameter, such as degree or centrality. These node-wise features were converted to pairwise features by taking the average distance for that feature between the nodes in each pair. Pairwise features were defined as those that apply to a pair of nodes such as shortest path or cohesion. The spin glass random walk features discussed below were included in our pairwise category. GO related features were derived from shared annotations between pairs of genes [41] (for a full list of features see Table 1).

**Table 1:** Names and descriptions of the node-wise and pairwise network parameters and GO term features used in Slant.

Name	Class	Description
Betweenness	Node-wise	The number of shortest paths in the entire graph that pass through the node.
Constraint	Node-wise	Related to ego networks. A measure of how much a node’s connections are focused on single cluster of neighbours.
Closeness	Node-wise	The number of steps required to reach all other nodes from a given node.
Coreness	Node-wise	Whether a node is part of the k-core of the full graph, the k-core being a maximal sub-graph in which each node has at least degree k.
Degree	Node-wise	The number of edges coming in to or out of the node.
Eccentricity	Node-wise	The shortest path distance from the node farthest from the given node.
Eigen centrality	Node-wise	A measure of how well connected a given node is to other well-connected nodes.
Hub score	Node-wise	Related to the concepts of hubs and authorities the hub score is a measure of how many well linked hubs the nodes is linked to.
Neighbourhood n size	Node-wise	The number of nodes within n steps of a given node for n of 1, 2, 5 and 6
Adhesion	Pairwise	The minimum number of edges that would have to be severed to result in two separate sub-graphs separating the source and target nodes.
Cohesion	Pairwise	The minimum number of nodes that would have to be removed to result in two separate sub-graphs separating the source and target nodes.
Adjacent	Pairwise	Whether a source and target node are connected via an edge.
Mutual neighbours	Pairwise	How many first neighbours a target and source node share.
Shortest path	Pairwise	The minimal number of connected vertices that create a path between the source and target node.
Between community	Pairwise	A logical feature stating whether the source and target nodes inhabit the same community produced by the spin glass random walk.
Cross community	Pairwise	A logical feature stating whether the source and target nodes connect two communities as produced by the spin glass random walk.
Shared GO count–Biological process	Go term	The number of biological process GO annotations shared between the source and target node.
Shared GO count–Molecular function	Go term	The number of molecular function GO annotations shared between the source and target node.
Shared GO count–Cellular compartment	Go term	The number of cellular compartment GO annotations shared between the source and target node.

**Fig 2:**
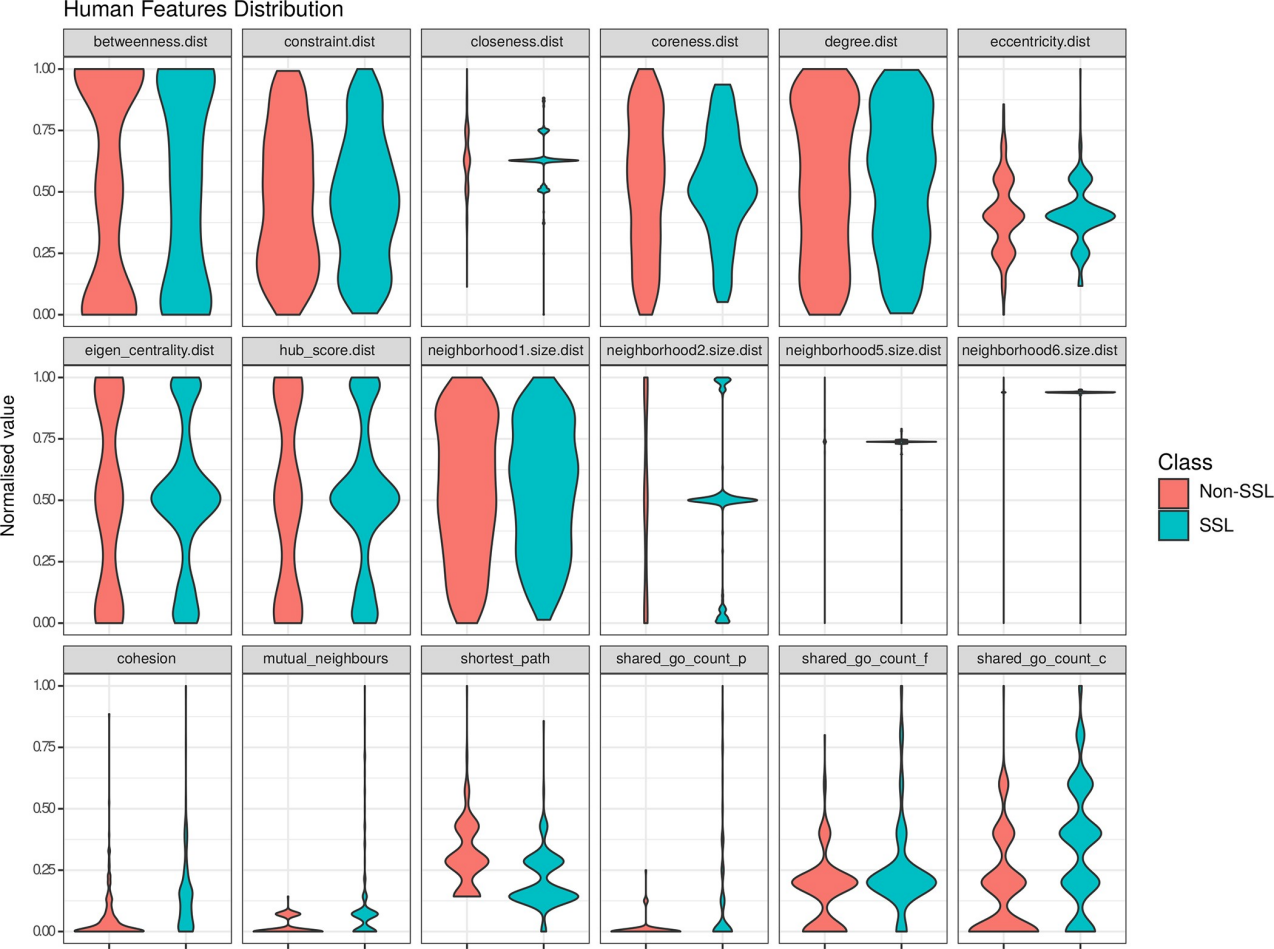
A set of violin plots illustrating the value distributions for each feature in our human training set grouped into SSL and non-SSL classes. The features were derived from 411 SSL and 411 non-SSL gene pairs (see S6 Table). Feature distributions that show greater variance between SSL and non-SSL gene pair classes, for example the shortest path feature, often provide improved predictive power in classifiers.

Fig 2 shows the distribution of these features in SSL and non-SSL gene pairs in humans. In general pairwise parameters showed a greater variance between SSL and non-SSL classes than our node-wise parameters suggesting they may prove better predictors in our models. Of these pairwise parameters the most notable differences were observed in the parameters labelled: cohesion—the minimum number of nodes that would have to be removed to result in two separate sub-graphs separating the source and target nodes, shortest path—the minimum number of nodes that must be traversed in a path between the source and target gene, and mutual neighbours—the number of nodes that are shared as neighbours between the source and target gene.

The higher values exhibited by gene pairs in the SSL class for the cohesion feature (paired t-test; p = 2.2e-16 in *H*. *sapiens*) suggest that SSL pairs are generally more densely connected in a physical interaction graph than non-SSL pairs (S1A Fig).

We also note that the shortest path between gene pairs is shorter on average for SSL gene pairs compared to non-SSL gene pairs (paired t-test; p = 4.589e-11 in *H*. *sapiens*) (S1B Fig) and, related to the shortest path parameter, SSL genes more often share a large number of mutual neighbours (paired t-test; p = 4.058e-11 in *H*. *sapiens*) (S1C Fig).

In terms of node-wise features it is of some interest to note that the difference between neighbourhood sizes of two genes in an SSL pair often differ more than those in a non-SSL pair.

### Random walk community generation suggests that most SSL interactions occur between rather than within clusters of genes

**Fig 3:**
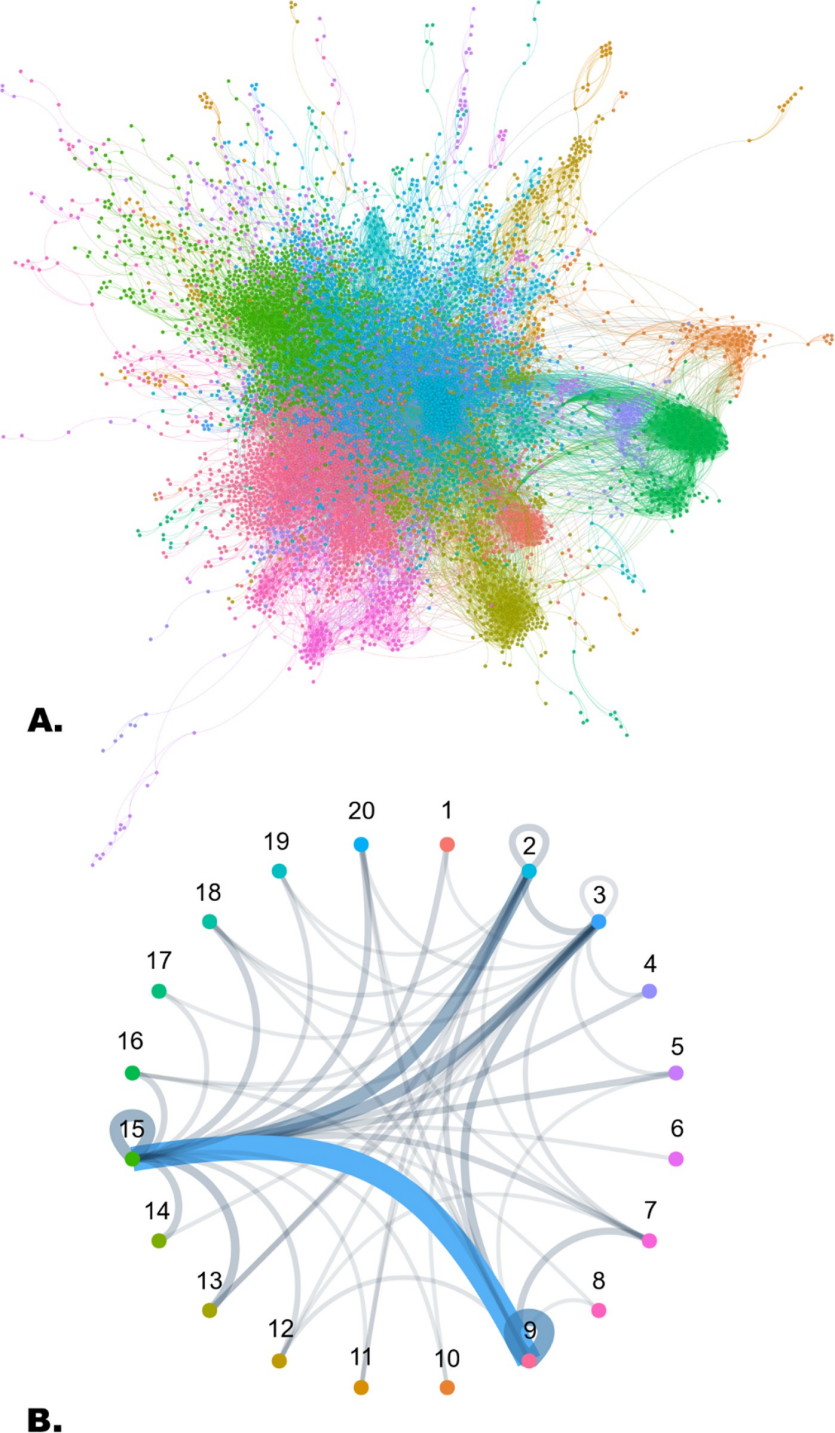
**a.** Human protein-protein interaction network with clustered communities generated by a spin glass random walk. Nodes and edges are coloured by their source community cluster as per the legend provided in Fig 3B. **b.** Community cluster connection graph where the weight of each connection corresponds to how many SSL interacting pairs begin and end at each community. We observe the largest count of SSL interactions occurring between cluster 9, notably associated with transcription regulation and DNA damage response GO terms and cluster 15, associated with MAPK cascade, cell proliferation and gene expression GO terms.

In an attempt to ascertain whether synthetic lethal interactions occurred within or between local clusters of genes in our physical interaction network we applied a spin-glass random walk to assign genes to 20 distinct communities separated by choke points across the graph (Fig 3A). Analysis showed that the majority of SSL interactions occurred between these communities rather than within (Fig 3B). In addition pairwise topological analysis suggests that SSL pairs of genes have shorter paths between them than non-SSL pairs and a higher occurrence of adjacency. Together these analyses suggest that SSL pairs are often at the peripheries of these communities, connecting their respective clusters.

Based on these observations we were able to create two additional features which provide further predictive power for classifying SSL pairs; whether nodes shared a community and whether the pair connected two communities.

### SSL pairs shared more GO annotations than non-SSL pairs

The count of shared GO terms, that is the number of GO annotations that two genes in a pair share with each other, also varies between SSL and non-SSL observations. SSL pairs generally share, on average, less biological process GO annotations (S1 Table) than non-SSL pairs (p < 2.2e-16 in *H*. *sapiens*) and proportionately more molecular function and cellular component GO annotations (p < 2.2e-16 in *H*. *sapiens* for both biological process and cellular compartment terms). This supports the view that that SSL protein product pairs are often found in similar but distinct pathways rather than within a single pathway [43]. Damaging two complementary functional pathways is likely cause more stress to the cell than damaging one pathway twice and leaving the complementary pathway functional.

Although the GO annotation based features above provide predictive power in our models as discussed below, due to the hierarchical nature of GO annotation, comparing the absolute count of shared GO terms does present some issues. As such GoSemSim [44] was used to further measure the semantic similarity between SSL and non-SSL pairs. We found that in *H*. *sapiens* SSL pairs showed a significantly higher semantic similarity score (mean = 0.65) that non-SSL pairs (mean = 0.57) (Welch two sample t-test p = 4.6e-07).

Analysis of GO terms present in paired SSL genes found that the most commonly shared molecular functional GO annotation related to protein binding (S2 Fig). Other molecular function GO annotations commonly found associated between SSL pairs include protein complex binding, GTP binding, DNA binding and GTPase activity. At the level of biological process GO annotation for SSL gene pairs we also noted associations with terms related to positive regulation of cell proliferation and negative regulation of apoptotic process as well as those labelled with positive regulation of gene expression and positive regulation of transcription from RNA polymerase II promoter.

In an attempt to further quantify the GO annotation driving the variation between genes found in SSL pairs and those not found in SSL pairs we employed a GO enrichment analysis using the on-line GOrrila tool [45]. We found significant enrichment in a number of GO annotations including negative regulation of cell differentiation (p = 9.15e-3), positive regulation of transcription by RNA polymerase II (p = 9.53e-3) and regulation of Notch signaling pathway (p = 8.85e-3) in the biological process ontology but no further enrichment in the molecular function or cellular compartments ontologies. All p-values have been are corrected for false positives using the Benjamini Hochberg method.

### SSL interactions in essential genes

Comprehensive studies of *S*. *cerevisiae* genetic interactions by Costanzo et al [37, 46] have found that essential genes that share an edge on the PPI network are enriched for genetic interactions and that is consistent with previous observations [43]. As our classifiers in part use the distance of gene pairs as a predictive feature we performed analysis to ensure our predictions were not simply picking out gene pairs enriched for essential genes.

We first noted that the range of shortest path values between SSL pairs on the protein-protein interaction (PPI) network runs from 1 to 7 with a mean of 2.43 and a standard deviation of 0.78 affirming that our training set features many SSL pairs that are not adjacent in the PPI network.

Using a set of essential human genes defined by Wang et al. [47], we found that 11% of the genes in our SSL training set were defined as essential, where as for non-SSL genes it only 0.7%. For human gene pairs ~1.7% of SSL pairs and ~1.4% of non-SSL pairs are comprised of two essential genes. We also found that 29% of SSL pairs and 22% of non-SSL pairs included at least one essential gene.

Upon comparison we found that ~22.5% of our SSL predictions included at least one essential gene and ~1.4% featured two essential genes, a ratio comparable with our training data. This suggests that our predictions are not further enriched for essential genes.

#### Models explaining patterns of genetic interactions

There are three models used to explain how genetic interactions occur [43, 48, 49]. The “between pathway model” is where the genetic interaction involves genes in two distinct pathways with complementary functions. A deletion of a gene in one pathway abrogates the function of that pathway and the cell cannot survive with of both pathways are lost. The “within a pathway model” is where genetic interaction occurs between genes in the subunits of a single pathway. Loss of one gene can be tolerated but the additive effects of the loss of several genes in that pathway are lethal. Finally 'the indirect model' is where the phenotype is not mediated by a localised mechanism.

Previous computational analyses have found that negative genetic interactions are enriched both between biological processes (or pathways) and within biological processes, giving credence to these models [37, 43, 46, 50]. SSL interactions occur primarily between local clusters in the PPI network suggest that the between pathways interactions may still involve pathways that are close in PPI space. This may explain why the analysis of PPIs is so effective in predicting SSL interactions.

### Network parameter distributions in model organisms

The distribution of network parameters across our four model organisms widely followed similar trends with our human feature set. Again the pairwise features for each organism appear to vary more between SSL and non-SSL classes than node-wise features. A few dissimilarities were noticeable, for example while SSL gene pairs tend to exhibit a higher levels of adhesion and cohesion in *H*. *sapiens*, *S*. *cerevisiae* (S3A Fig) and *D*. *melanogaster* (S3B Fig) the distribution for these features were notably inverted in *C*. *elegans* (S3C Fig) and *S*. *pombe* (S3D Fig) so that non-SSL pairs showed higher adhesion and cohesion than SSL pairs.

### Validating SSL gene pair classification

In this study we perform two classifications. First in-species classification, classifying and validating SSL gene pairs using training and test data from the same organism. Then cross-species classification where we use the models built using the training data for each organism to blindly predict SSL for each other species. Within each species, the feature data were normalised and segmented into training and test sets with 20% set aside for validation. We employed 5-fold cross validation to optimise the hyperparameters for each organism's random forest classifier and evaluated in-species classification performance (Table 2). In this study our random forest classifiers utilised just one hyper-parameter, mtry—the number of variables randomly sampled as candidates at each split for each tree. The best classifier for each species was then used to predict the SSL gene pairs in each of the other four species. Table 2 shows the ROC AUC scores for both the in-species and cross-species predictions for all of our models.

**Table 2:** Cross validation ROC AUC scores for each organism from both in-species and cross species SSL models. The best score for each species model is highlighted in green. Models are displayed vertically in rows with the consensus model displayed at the bottom of the table and the results for those models are displayed in columns with the consensus results highlighted in blue.

		Validation results				
		H. sapiens	S. cerevisiae	C. elegans	D. melanogaster	S. pombe
**Model**	H. sapiens	0.965	0.698	0.662	0.687	0.661
	S. cerevisiae	0.713	0.883	0.694	0.784	0.717
	C. elegans	0.769	0.598	0.979	0.744	0.588
	D. melanogaster	0.727	0.790	0.816	0.906	0.778
	S. pombe	0.48	0.607	0.574	0.660	0.889
	Consensus	0.985	0.907	0.982	0.903	0.920

Although it is difficult to compare the performance of classifiers due to varied validation sets, the ROC AUC score of 0.965 for *H*. *sapiens* SSL gene pair classification achieved by the SLant classifier (using holdout validation data) appears to out-perform Daisy’s ROC AUC score of 0.779.

Our initial in-species classification of *S*. *cerevisiae* SSL resulted in relatively low performance (AUC 0.734) compared to other related studies. For example MetaSL, which used a much smaller data set of just 7,347 SSL pairs compared to Slant’s 395,199 pairs, achieved ROC AUC scores of up to 0.871 [33]. In order to mitigate any noise or error introduced in our large dataset we filtered out any SSL interactions reported in BioGRID supported by less than 3 supporting publications for *S*. *cerevisiae* and less than 2 papers for *S*. *Pombe*. Our training data ultimately used 17,568 out of a total 395,199 SSL pairs available for *S*. *cerevisiae* and 3,836 out of 35,391 SSL pairs for *S*. *Pombe*. These sample sizes should still be large enough to generalise well for out of sample predictions as well as preforming well in classification and validation. Filtering our yeast data improved our scores from AUC ROC 0.734 to AUC ROC 0.883 for *S*. *cerevisiae* and 0.728 to 0.889 for S. *Pombe* which suggests that by removing pairs that show fewer citations in the BioGRID data we are reducing variation in our training data introduced by false positives. This may be due to the relatively high false-positive rate found in large scale GI screenings, an observation supported by analysis performed by Campbell & Ryan et al. who estimated that large scale screenings can suffer a false positive rate of up to ~10% [51]. Using this value we can calculate that by removing GI pairs with less than 2 and 3 references respectively we may be reducing false positive rates from 1/10 to 1/100 in *S*. *pombe* and from 1/10 to 1/1000 in *S*. *cerevisiae*.

Cross-species predictions of SSLs were quite variable in performance. Models from both *S*. *cerevisiae* and *D*. *melanogaster and C*.*elegans* were successful in predicting human SSLs with AUC ROC scores of 0.713, 0.727 and 0.769 respectively.

Although the *C*. *elegans* classifier performed relatively poorly in our cross-species validation for *H. sapiens* classification, this variation may help improve the generalisation of our consensus model which is discussed below. To test this cross-species validation was performed without the worm model. The removal of worm data from the classifier resulted in a small but noticeable decrease in performance of the consensus classifier for humans (decreasing from ~0.985 to ~0.92).

The result here suggest that the PPI patterns between SSL genes are similar both within and between species and that network topology features used in our classifiers generalise well across organisms. We identified the most predictive features for each organism and found that the same features were most predictive in many of the species. The shared GO count features were important in all organisms except *S*. *pombe* and the pairwise features adhesion, cohesion, mutual neighbours and adjacency were important in all organisms except *C*. *elegans*. Two node-wise features, coreness and neighbourhood size are also listed as important features across 3 organisms (S2 Table).

### Class balance changes do not significantly impact classifier performance

As described below in methods each of these models use a balanced training set with a ratio of 1:1 interacting and non-interacting pairs, however in reality the ratio between interacting and non-interacting pairs is likely more in the order of 1:50 based on global yeast GI screens [37]. To ascertain that our class balance has not unduly biased our prediction in any way we re-ran our classifiers using a randomised training / validation set with approximately 1:10 and 1:50 class balance. We found that with a class balance of 1:10 our performance remained stable and with a class balance of 1:50 we found just a small drop in performance (human AUC ROC ~0.87 compared to the original ~0.965 and consensus AUC ROC ~0.90 compared to ~0.985).

### Our models are robust to incompleteness in the source PPI networks

It is known that our current PPI models are incomplete [52–54] and suffer from ascertainment bias. That is, some genes, and indeed some species, are better studied than others. To test our model’s robustness to the incomplete nature of the protein-protein interaction networks, we re-ran our classifiers holding out 10% and 20% of the nodes, at random, from original PPI data in *H*. *sapiens*. In the case of the 90% ‘complete’ PPI network the performance of our in-species model validation was not effected and our *H*. *sapiens* consensus showed just a small drop in performance (from AUC ROC ~0.985 to ~0.922). With a 80% ‘complete’ *H*. *sapiens* PPI network we saw another fairly small incremental drop in *H*. *sapiens* consensus performance (AUC ROC ~ 0.85) and a small drop in *H*. *sapiens* in-species performance (AUC ROC dropping from 0.965 to 0.911). This suggests both that an increasingly complete PPI network may incrementally improve our predictive performance and that the current models are fairly resilient to the incomplete nature of the PPI network.

### Our pair-wise distance features are the most predictive

In addition to the feature importance analysis performed in this study we also re-ran our classifiers holding out our 12 node-wise distance features, 6 pair-wise features and 3 GO-term related features in turn. We found that the model holding out pair-wise features saw the largest drop in performance in consensus with the *H*. *sapiens* consensus ROC AUC dropping from ~0.985 to ~0.730 and the in-species *H*.*sapiens* ROC AUC dropping from ~0.965 to ~0.82. In comparison to our models holding out node-wise features saw a more notable drop in the in-species performance (*H*.*sapiens* consensus ROC AUC dropping from ~0.985 to ~0.85 and in-species *H*.*sapiens* from ~0.965 to ~0.823). Similarly holding out our GO term features resulted in a decrease in predictive performance (*H*.*sapiens* consensus ROC AUC dropping from ~0.985 to ~0.882 and in-species *H*.*sapiens* from ~0.965 to ~0.890).

### Our models are moderately robust to pair-input bias

As discussed by Parks et al. [55] computational prediction methods that utilise gene pair observations, such as the models in this study, can be subject to positive bias in validation. They discovered that model validation performed significantly better when genes that made up the pairs in the test set were also featured in the training set compared to those models where they were not.

In order to evaluate how SLant’s validation was effected by pair-input bias we generated a test set from our raw feature data in which none of the genes featured in the test pairs were present in any of the pairs featured in the training set. We refer to these as segregated datasets.

To make sure we could make a fair comparison we generated a further control training and test set by randomly sampling the pairs created above from both segregated data sets. This ensured that the pair count and the pairs themselves remained the same while gene components could be shared between our control training and test sets.

Running our models again using these segregated training and test data we achieved a AUC ROC of 0.789 for predicting human SSL pairs, compared to 0.845 for our control datasets and 0.965 for our full training and test sets. This suggests that while our predictions may be somewhat biased towards genes that are featured in the training data our models also appear to predict SSL pairs comprised of genes that are not in our training data and, more importantly, potentially genes that have not previously been associated with SSL interactions.

### A consensus based on many cross-species predictions further improves performance

To further expand our model we took a consensus from the cross-species predictions for each organism. This consensus was calculated by running a second classifier, a boosted general linear model (GLM) that was trained on the previous cross-species classifier output. This output took the form of confidence scores. For example, for any particular pair of human genes the confidence scores given to that pair by every cross-species classifier were used as features. The probability outputted by this final classifier is referred to as the consensus score.

**Fig 4:**
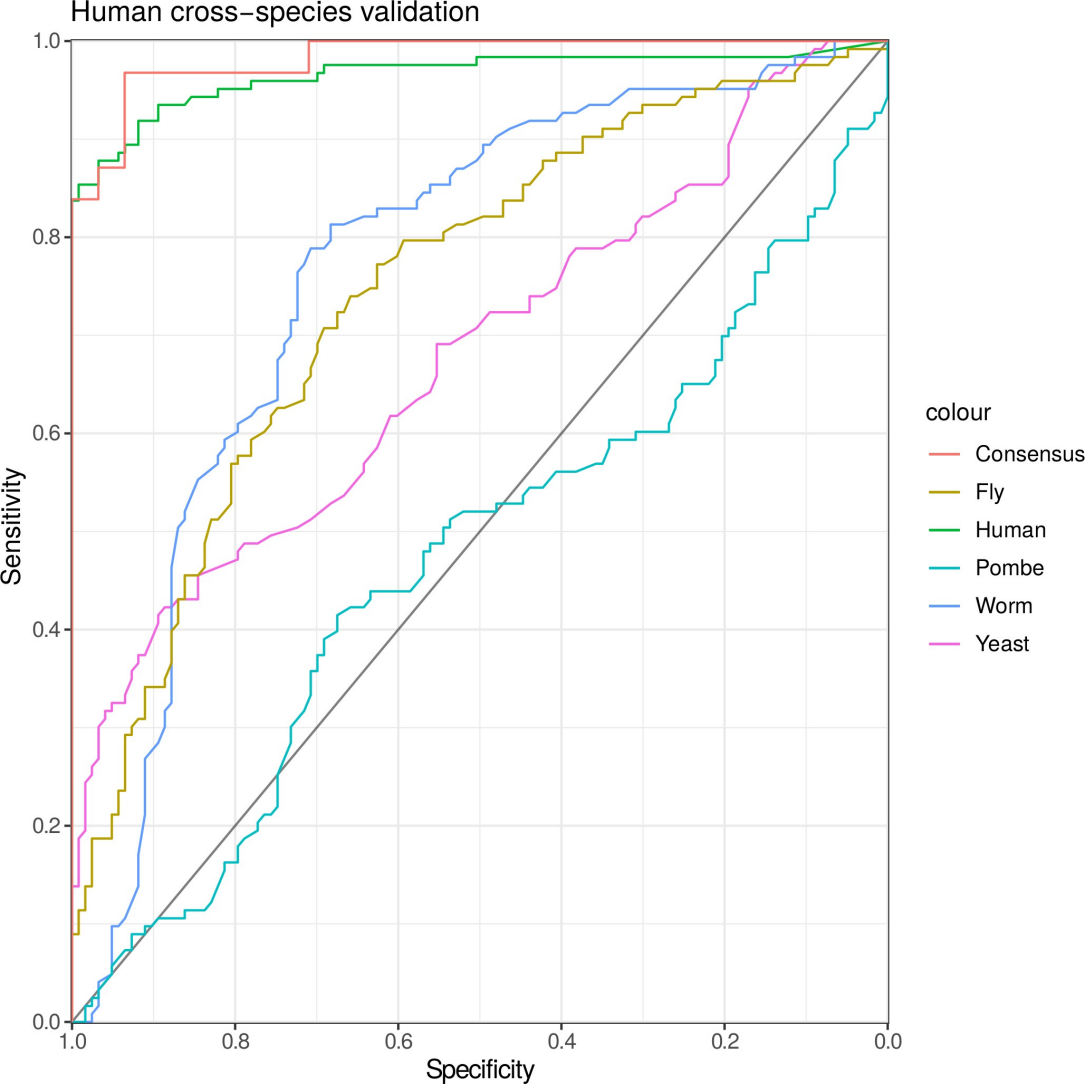
Cross-species ROC AUC scores for each models classification performance on our human SSL interaction validation set. An additional curve for our consensus predictions was added separately based on the performance of the consensus validation set.

To allow for validation this consensus dataset was segmented into a training and test set (both 0.5 the size of the original due to the smaller overall size). The ROC AUC for our consensus prediction validation was also plotted and achieved a score of up to 0.985 when predicting *H*. *sapiens* SSL pairs, a further improvement on our in-species human validation ROC AUC score of 0.965 (Fig 4).

### Predicting synthetic dosage lethal pairs

To ascertain whether SSL and synthetic dosage lethality (SDL) interactions share topological predictors we re-purposed our models to predict SDL gene pairs. We achieved an in-species AUC of 0.78 for *H*. *sapien*s pairs and 0.89 for *S*. *cerevisiae* pairs, a significantly improved score compared to that achieved during *S*. *cerevisiae* SSL pair classification. Our consensus model, utilising just *H*. *sapiens* and *S*. *cerevisiae* data, improved our *H*. *sapien*s predictions slightly (ROC AUC 0.80) (S3 Table).

SDL and SSL pairs in *H*. *sapiens* exhibit broadly similar feature distribution and feature importance for both classifiers. Despite this only 7,531 pairs were predicted as both SDL and SSL (of 41,103 SDL pair predictions and 59,475 SSL pair predictions).

In our human SDL models cohesion and shared cellular compartment GO terms featured as important features for both classifiers though molecular functional GO term annotation proved an important feature for SDL classification while shared biological process GO term featured well for SSL classification. The closeness feature, which measures how many steps is required to reach all other nodes from a given node, performed well for SDL classification. On the other hand coreness, a measurement of how well connected a node's neighbours are compared to the graph overall provided better predictive power for SSL classification.

We next compared biological process GO terms present in SDL and SSL pairs. We found that DNA damage related processes were more frequently seen in SDL pair data than in SSL pair data (~1.00% cellular response to DNA damage stimulus, ~0.70% DNA repair in SDL pairs compared to ~0.53% and ~0.46% respectively in SSL pairs). MAPK cascade and regulation of cell proliferation processes were well represented in both groups.

### Comparison to previous studies

As discussed in the introduction, a number of other studies have used similar methods to predict genetic interactions. Most notably, this study shares a number of similarities with SINaTRA [32]. However, SLant has been developed for a wider number of organisms, including using human data directly, uses an enhanced feature set, our predictions have been experimentally validated (see below) and all of our data are available via the SLorth database (see below).

Algorithmically, the similarities between SLant and SINaTRA include some of the features used and the treatment of normalisation to allow cross-species prediction. However the PPI data used by SLant were sourced from STRING and were filtered for reliability, while SINaTRA's PPI data were sourced from BioGRID. A number of key algorithmic differences include SLant's use of consensus models, for both SSL and SDL interactions, and the use of a large range of topological, community and GO features. SLant also treats node-wise features differently and includes the averaged difference between genes in a pair as well as the individual values for each gene. We show that the novel features present in SLant improve the results in the feature holdout section (see *Our pair-wise distance features are the most predictive*) and propose that the different data sets appear to be providing a large impact on the results. A comparison of the features used in the two studies are available in S7 Table.

Unfortunately, the source code for SINaTRA is not available. However we were able to assess how our algorithm performed compared to SINaTRA, by testing it on the historical yeast SSL data from BioGrid 3.2.104 that had been used in the development of the SINaTRA algorithm. SINaTRA reports impressive AUC ROC values of 0.92 for in-species *S*. *cerevisiae S*SL predictions, 0.93 for in-species *S*. *pombe* SSL predictions, 0.86 for *S*. *cerevisiae* to *S*. *pombe* cross species validation and 0.74 for *S*. *pombe* to *S*. *cerevisiae* cross species validation. We obtained similar results using cross validation (as reported by SINaTRA) with AUC ROC values of 0.98 for in-species *S*. *cerevisiae* SSL predictions, 0.98 for in-species *S*. *pombe* SSL predictions, 0.88 for *S*. *cerevisiae* to *S*. *pombe* cross species validation and 0.77 for *S*. *pombe* to *S*. *cerevisiae* cross species validation (see S8 Table).

Next, we re-implemented SINaTRA by running SLant with a close approximation of the features that SINaTRA used originally but using the current STRING PPI network and current SSL data for training (see S9 and S10 Tables). We found that SLant outperformed SINaTRA in all tests apart from the *S*. *pombe* to *S*. *cerevisiae* cross species validation (AUC ROC 0.607 versus 0.609). In particular SLant considerably outperforms SINaTRA using models generated using the pair-wise non-bias segregated training sets. This supports our theory that the additional pairwise features incorporated into SLant leads to a generalisation of the models.

Finally we analysed the 2518 predicted human SSL pairs, with a SINaTRA score of over 0.90, that were published in the original paper. Of these, none of these predictions have subsequently been reported in BioGRID, either as SSLs or as negative genetic interactions. However, the number of reported SSLs for humans is still rather low. Encouragingly, 55% of the SINaTRA high confidence SSL predictions were also predicted to be SSLs by SLant.

### Slorth database

We employed the full cross-species consensus model to predict SSL and SDL gene pairs in all of our species. All pairs that did not achieved a consensus score of over 0.75 were removed from our final prediction list. All predictions are available in the Slorth database http://slorth.biochem.sussex.ac.uk.

The graphical visualizations of the SSL predictions and the experimentally derived SSL interactions from our training data (S4A Fig) shows that the SSL network becomes much denser around the genes represented in the initial training data from BioGRID. This suggests that genes already implicated in an SSL pairs may share more SSL interactions than currently experimentally identified.

### Predicting and validating SSL gene pairs associated with cancer

Using the models and classifiers described above we have identified and validated previously unpublished human SSLs that could be exploited therapeutically in the treatment of cancer. To identify potential therapeutic targets using our consensus method, we identified all the SSL gene pairs in *H*. *sapiens* where one of the genes had been identified as a tumour suppressor by the cancer gene census [56] (S4B Fig, appendix Table 4) and the other was a target of a drug approved for human use.

We found an enrichment in highly scoring SSL pairs containing the tumour suppressors *VHL* and *PTEN*. SSL pairs with the highest consensus scores included *SREBF1*, a transcription factor that binds to sterol regulatory element-1 and *VHL* (confidence score 0.810) and *PTEN* and *SFN*, a gene associated with breast cancer (confidence score 0.808). Other novel, highly scoring gene pair predictions that included cancer associated genes included *PARP1* with *PBRM1*, *BRCA2*, *ARID1A* and *APC* as well as *PIK3CA* with *MAP2K1*, *ABL1* and *EGFR*.

*Va*lidation on a handful of these predicted pairs providing some evidence that *PBRM1* / *PARP1* and *PBRM1* / *ABL1* share previously undescribed SSL interactions. We also see some evidence that PBRM1 / POLA1 share a synthetic rescue interaction.

### Experimental validation of predictions

A set of predicted gene pairs, where one of the genes identified was *PBRM1*, was selected for experimental validation. The *PBRM1* gene codes for the tumour suppressor BAF180 a protein that plays a key role in both chromatin remodelling and gene transcription. It is frequently mutated in a subset of cancers including Clear Cell Papillary Renal Cell Carcinoma and Clear Cell Renal Cell Carcinoma [57] We chose gene pairs where the second gene codes for a protein which has published inhibitors. These included; *PARP1*, *ERBB2*, *RAF1*, *POLA1*, *JAK2*, *ABL1*, *GSK3B* (S5 Table). Inhibitors were chosen and procured via Sellekchem (https://pubchem.ncbi.nlm.nih.gov/source/Selleck%20Chemicals).

Clonogenic survival assays [58] were prepared for a control group and a BAF180 knockout group from the U2OS cell line. Both cell groups were treated with a range of drug concentrations based on previous literature for each. The resulting cell colonies were stained and counted after 14 days of incubation.

**Fig 5:**
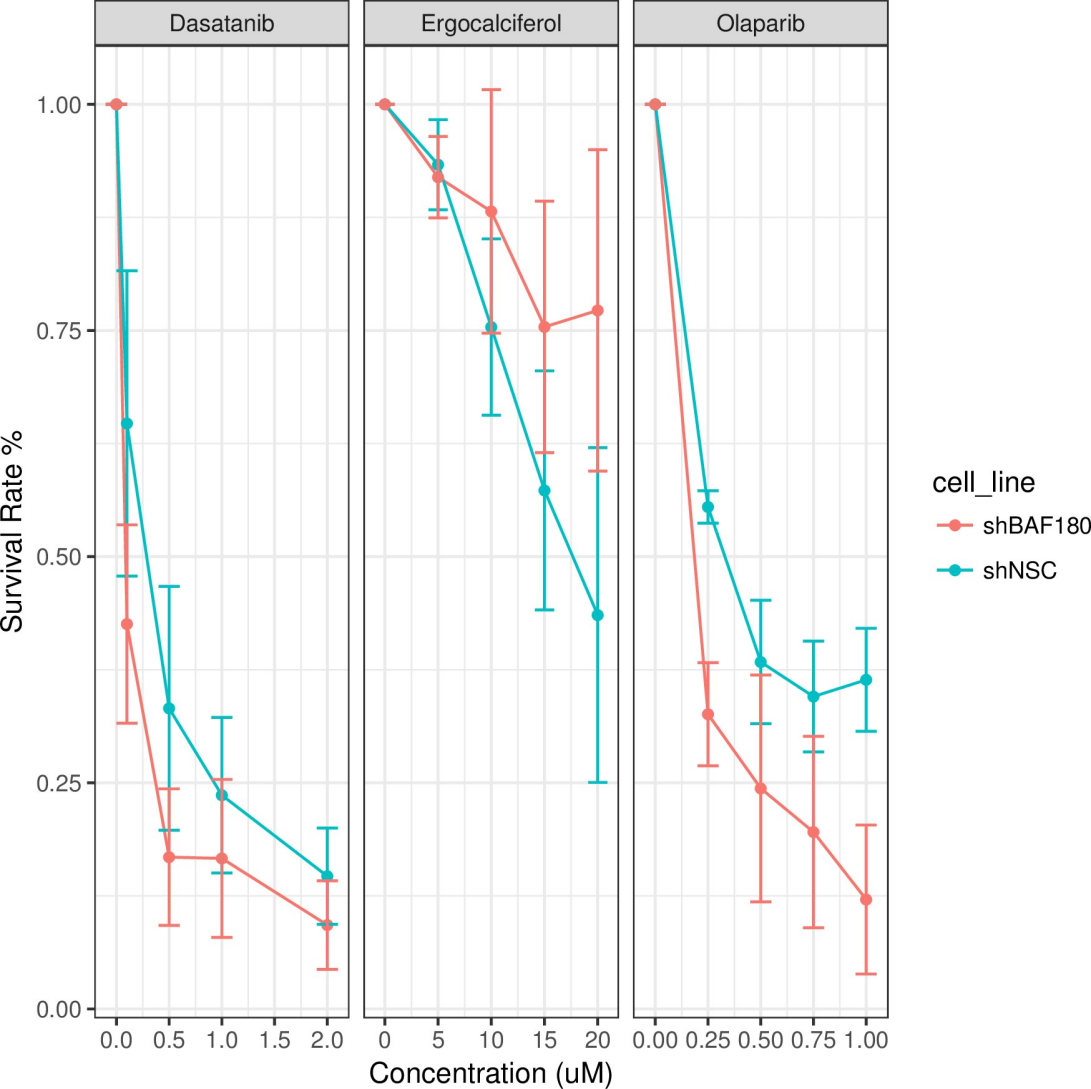
Carcinogenic survival assay results charting survival of PBRM1 / BAF180 knock-out cell lines with concentration intervals of the PARP inhibitor Olaparib, the POLA inhibitor Erocalciferol and the ABL inhibitor Dasatanib. These results suggest PBRM1 mutant cells may be more sensitive to both the PARP and ABL1 inhibitors while gaining some resistance to POLA1 inhibition. Error bars measure standard error of measurement. All drug intervals are measured in mM.

Of the drugs tested, three showed differential effects on the BAF180-deficient cells when compared to the control cells. PBRM1 mutant cells were more sensitive to both the PARP inhibitor and, to a lesser extent, ABL1 inhibitor than the control cells (Fig 5 with plate photography in S5 Fig), whereas the PBRM1 mutant cells appeared less sensitive to the POLA1 inhibitor than the control cells (Fig 5). Interestingly, cells lacking ARID1A, which is another SWI/SNF subunit, are also selectively sensitive to PARP inhibitors [59, 60], which supports this relationship. We also note this ARID1A / PARP1 SSL interaction was not present in the BioGRID data used to generate our training set but was also predicted with a high probability by SLant. The two protein products of the two genes SSL with *PBRM1*; *PARP1* and *ABL1*, share a number of similar cellular processes such as regulation of differentiation, proliferation and of DNA damage and stress response. POLA1 which potentially shares a different type of interaction, synthetic rescue, plays an essential role in the initiation of DNA replication.

## Discussion

In this paper we have predicted SSL relationships using features derived from both in-species and cross-species PPI network information. The SLant consensus classifier out-performs previous attempts at predicting human and model organism SSL interactions and may provide a useful tool in guiding future experimental validation of SSL pairs.

The original intention in this study was to predict cross-species without using the target species' data in the training set. However our in-species predictions generally performed so well it seemed sensible to instead use the additional cross-species data as an enhancement instead. The only in-species classifier that underperformed was that derived for *S*. *cerevisiae*. However, this result should be interpreted with caution; direct comparison of results is not possible as there are differences in the validation data. So that others may compare their algorithm to ours we have made all of the source code for SLant freely available so that our results, training data and validation can easily be recreated and repeated.

Improving the quantity and the quality of the input data will also improve the quality of the SSL and SDL predictions. For instance the amount of genetic interaction data is very limited in humans and *D*. *melanogaster*. Protein-protein interaction data is plentiful for humans and the model organisms studied, but the majority of the interactions are unlabelled. Adding additional annotation to these interactions, e.g. the direction of an interaction, may improve predictions if enough labelled data were available. Also, both the PPI and the genetic interactions reported have ‘popularity bias’; genes and proteins of biological or medical interest are frequently studied and hence more interactions involving them are reported.

Recently Abdollahpouri et al. [61] developed a flexible regularization-based framework which can be used to control for popularity. An adaptation of this method to enhance the coverage of less frequently reported genetic interations, may help mitigate this bias. Furthermore, providing a reliability score for genetic interactions and only using the more reliable ones may be particularly important for *S*. *cerevisiae* where although there is a wealth of data, the number of false positives reported experimentally may be corrupting the prediction accuracy.

In an attempt to ascertain whether synthetic lethal interactions occurred within or between local clusters of genes in our physical network we applied a spin glass random walk to assign genes to distinct clustered communities separated by choke points across the graph. Analysis showed that the majority of SSL interactions occurred between these communities rather than within them. Based on the shorter distance between SSL genes and higher occurrence of adjacency presumably SSL genes are often at the peripheries of these communities. Further exploration of how SSL pairs are distributed between clustered communities such as these may shed further light on the node wise features of genetic interactions.

Although this study does not use orthology data directly we do note that our GO annotation features may in some way serve as a proxy for orthology data and this study could be also be expanded in the future through improved analysis of the relationship between GO terms and pairwise SSL pairs.

The identification of SSL interactions is a key step in expanding and improving targeted cancer therapy. The results presented here suggest that inhibition of PARP1 or of ABL protein kinase 1 may have therapeutic value in tumours lacking functional BAF180. The computational and experimental validation of our models performance presented in this study suggests that the predictions provided by SLant, all of which have been made publicly available, will be useful in guiding future SSL screening studies and ultimately in the continued goal of generating a more complete list of human SSL pairs.

## Materials and methods

### Data Acquisition and pre-processing

Gene and orthology data were downloaded from Ensembl [62], Genetic interaction data were obtained from BioGRID (version 3.4.156) [42] with supplementary *D*. *melanogaster* data downloaded from Flybase (version 6.13) [63]. Each gene was labelled with gene ontology (GO) data from the gene ontology consortium [41]. Protein-protein interaction (PPI) data were obtained from the STRING database (version 10) [39]. To ensure reliability only experimentally derived and curated pathway data with a reliability cut-off of 80 were utilised (S6 Table). The Ensembl ENSP protein IDs in the PPI data sets were converted to their respective Ensembl ENSG gene IDs. This enabled us to relate the physical interaction data to the genetic interaction data and label each physical interaction gene pair as SSL (if present in the BioGRID data) or non-SSL (if the pair was not present in the BioGRID data).

For each organism an equal number of non-SSL pairs were assigned randomly to constitute the negative training set. When assigning a non-SSL pair, we checked to makes sure that its orthologues had not been assigned as having an SSL as, although it is not prescriptive, there is an enrichment of SSL pairs in orthologous genes.

Similar methods were used to build the training set used for our SDL interaction classifiers but we instead extracted BioGRID pairs annotated as synthetic dosage lethal as our positive class data.

### Feature processing

The R (version 3.4.0) igraph package (version 1.1.2) [40] was used to generate a network representation of the PPI data for each of our 5 organisms and to calculate network features. (Table 1). Whilst we extracted network features for just a subset of all possible gene pairs the entire network of protein interactions was used in each calculation.

The features generated for our models were broadly categorised as node-wise or pairwise features as listed in Table 1. In general node-wise features, such as degree, were calculated by extracting network parameters for single nodes and finding the averaged distance between them as a pairwise feature. Pairwise features such as shortest path were calculated by igraph on each pair. To calculate shared GO terms, classed as a pairwise feature, we took a count of overlapping GO terms between the genes in each pair.

To generate our community features we applied a spin-glass random walk using the R igraph communities module to assign genes to 20 distinct communities separated by choke points across the graph. The final count of communities, 20, was chosen by measuring the predictive performance of our community features with a community count incrementing in steps of 5. After 20 communities we saw no further improvement.

The entire feature generation pipeline for the full complement of available gene pairs proved computationally intense, especially the generation of pairwise features such as cohesion, and run-time took up to 120 hours for each organism on an 8x Intel Xeon 3.50GHz processor with 16Gb RAM.

### Training and test sets

Before analysis all features in each dataset were normalised so that all feature values fell between 0 and 1. The resulting feature sets were divided into training, test and unlabelled sets. For each organism the feature set was under sampled to provide a balanced training set with an equal number of SSL and non-SSL pairs. The training set was further partitioned 80:20 to create a test set. The non-SSL pairs removed from the training data as part of under sampling were set aside as unlabelled data to be used in the prediction section of this study.

### Creating balanced training and test pair sets with distinct gene components

Some genes are highly represented in our available SSL training data whilst some only occur once, so generating two sets with balanced classes and a requisite number of observations posed a challenge. To create balanced training and test datasets with enough observations to perform validation we first created a list of genes ranked by the number of pairs they were found in. Next we divided this list adding the first to our list of genes available in our training data, the second to our test data and so on so that both data sets had a similar distribution of gene representation. Finally we used these two gene lists to filter our feature data into two subsets with no overlapping genes and balanced class.

### Analysis and modelling

We used the “ranger” e1071 random forest classifier, part of the R caret library, to model and classify SSL and non-SSL interactions in our training set. 5-fold cross validation was applied to each organism's training set to tune the model's hyper-parameters and the best model was used to assess predictive performance within each species. These optimised models were then used to predict SSL pairs across species, both in *H*. *sapiens* and across all other model organisms. These predictions were outputted as the probability of each class and were validated against the test data set.

### Calculating cross species consensus

In an attempt to further improve accuracy, as well as pairwise cross-species predictions, a consensus was taken from the predictions on the test set from all other species. This consensus was calculated by running a second classifier, a boosted Generalized Linear Model (GLM) that was trained on the previous classifiers outputs. To allow for validation this consensus dataset was segmented into a train and test set (both 0.5 the size of the original due to the smaller overall size). Finally we used this consensus model to predict SSL pairs in the unlabelled data set.

All of the R source code for SLant is available publically at https://bitbucket.org/bioinformatics_lab_sussex/slant. All data used is available via public resources.

### Validation using clonogenic survival assays

A subset of potential SSL interacting pairs featuring *PBRM1* (BAF180) complemented with genes with a known inhibitor were chosen from our predictions for experimental validation (S5 Table).

#### Cell culture

U2OS-derived control and PBRM1-deficient cell lines [64] were cultured in Dulbecco DMEM supplemented with 10% FBS, glutamine and Penicilin/Streptomycin.

#### Clonogenic survival assays

Cells were seeded and allowed to adhere prior to drug treatment. Cells were exposed to the indicated amount of drug in triplicate, and incubated for 14 days at 37C with 5% CO2 prior to staining with methylene blue ((0.4%). Cell colonies were manually counted and presented as the surviving fraction relative to the untreated cells.

## Supporting information

S1 Fig Feature distributions. **a.** A distribution of normalised adhesion scores for each organism illustrate significant differences in SSL and non-SSL pairs across species. **b.** A normalised shortest path distribution shows a general trend for shorter shortest paths between *H*. *sapiens* SSL pairs though this difference is less pronounced in our model organisms. **c.** A distribution of normalised mutual neighbour counts suggests that SSL pairs often share more mutual neighbours than non-SSL pairs.
(TIFF)

S2 Fig GO terms. Count of most common associations between molecular function GO terms observed in SSL pairs. Individual feature GO associations extracted from full GO annotation lists for each SSL gene pair.
(TIF)

S3 Fig Feature value distributions. Violin plots illustrating feature value distributions for **A,** *S*. *cerevisiae*, **B,** *C*. *elegans*, **C,** *D*. *melanogaster* and **D,** *S*. *pombe*.
(TIFF)

S4 Fig SSL interaction networks. **a.** Full SSL interaction network of predicted human SSL pairs shaded by likelihood of being a true SSL pair based on consensus score. Red edges are interactions sourced from our training data (directly from BioGRID) lighter edges denote a lower consensus scores. Produced with Gephi 0.9.1 [8]. **b.** Network of SSL interaction predictions with high consensus scores associated with known tumour suppressors including, where available, VHL, BRCA1, BRCA2, PBRM1, PTEN and APC.
(TIFF)

S5 Fig Survival assay plate images. **S**urvival assay plate images for ABL inhibitor Dasatanib (marked as Dasat) (**A**, **B**, **C** & **D**) and POLA inhibitor Erocalciferol (marked as VD2, an abbreviation of vitamin D2) experiments (**E**, **F**, **G** & **H**). BAF180 knock-out cell-line plate images for the PARP1 inhibitor Olaparib BAF180 are labeled with BAF and control plates marked with NSC on plate lids and the corresponding plate colonies are displayed adjacent to each lid (**I** & **J**).
(TIFF)

S1 References (DOCX)

S1 Table Distribution of shared molecular function, biological process and cellular compartment GO terms that occur between SSL and non-SSL pairs. Data is shown for **A,** *H*. *sapiens*, **B,** *S*. *cerevisiae*, **C,** *C*. *elegans*, **D,** *D*. *melanogaster*, and **E,** *S*. *pombe*. We observe that in humans SSL pairs share significantly more molecular function and cellular compartment GO terms while non-SSL pairs share significantly more biological process terms. A welch 2 sample t-test was used to measure significance for each annotation. 2.2e-16 was the smallest value available.
(DOCX)

S2 Table Key features. This table contains a list of most important features for each species reported via the R caret libraries random forest classifier. Feature importance rankings were calculated by measuring the mean decrease in accuracy without each variable across all tree permutations in the random forest.
(DOCX)

S3 Table Cross validation ROC AUC scores for S. *cerevisiae* and H. *sapiens* SDL models. The best score for each species model is highlighted in green. Consensus model results are highlighted in blue.
(DOCX)

S4 Table Top 20 SSL predictions featuring common tumour suppressor genes. (DOCX)

S5 Table Genes SSL with BAF180. We chose a group of genes with selective inhibitors that were predicted to share a synthetic lethal interaction with BAF180 (PBRM1) for validation. We performed clonogenic survival assays for each inhibitor using U2OS cell lines (shControl + mCherry/NLS and shBAF180 + GFP/NLS).
(DOCX)

S6 Table Number of protein-protein interactions used to generate the protein interaction networks for each organism. Number of SSL pairs and SDL pairs sourced for each organism from BioGRID after filtering for distinct pairs that inlcude genes present in the protein interaction network. The SSL pair data for *S*. *cerevisiae* were filtered to include only interactions cited in 3 or more papers. SSL pair data for *S*. *pombe* were filtered to include only interactions recorded in 2 or more papers.
(DOCX)

S7 Table A comparison of the features used by SLant and SINaTRA. SLant also treats node-wise features differently by providing an averaged difference between node pairs as well as the individual values per gene node.
(DOCX)

S8 Table A comparison of SLant and SINaTRA AUC ROC scores using SSLs from *BioGRID* 3.2.104. SLant data were generated in house, SINaTRA scores were extracted from Jakunski et al., 2015 publication.
(DOCX)

S9 Table A comparison of classification performance. AUC ROC scores from the SLant feature set versus the SINaTRA feature set using the full current training sets and the pairwise non-bias data sets.
(DOCX)

S10 Table A comparison of classification performance. A comparison of human SSL classification using the SLant consensus set versus the SINaTRA feature set using current data.
(DOCX)
