# Supplementary figures and images for: Predicting synthetic lethal interactions using conserved patterns in protein interaction networks
Source: PLoS Comput Biol. 2019 Apr 17;15(4):e1006888. doi: 10.1371/journal.pcbi.1006888 (PMC6488098; doi:10.1371/journal.pcbi.1006888)

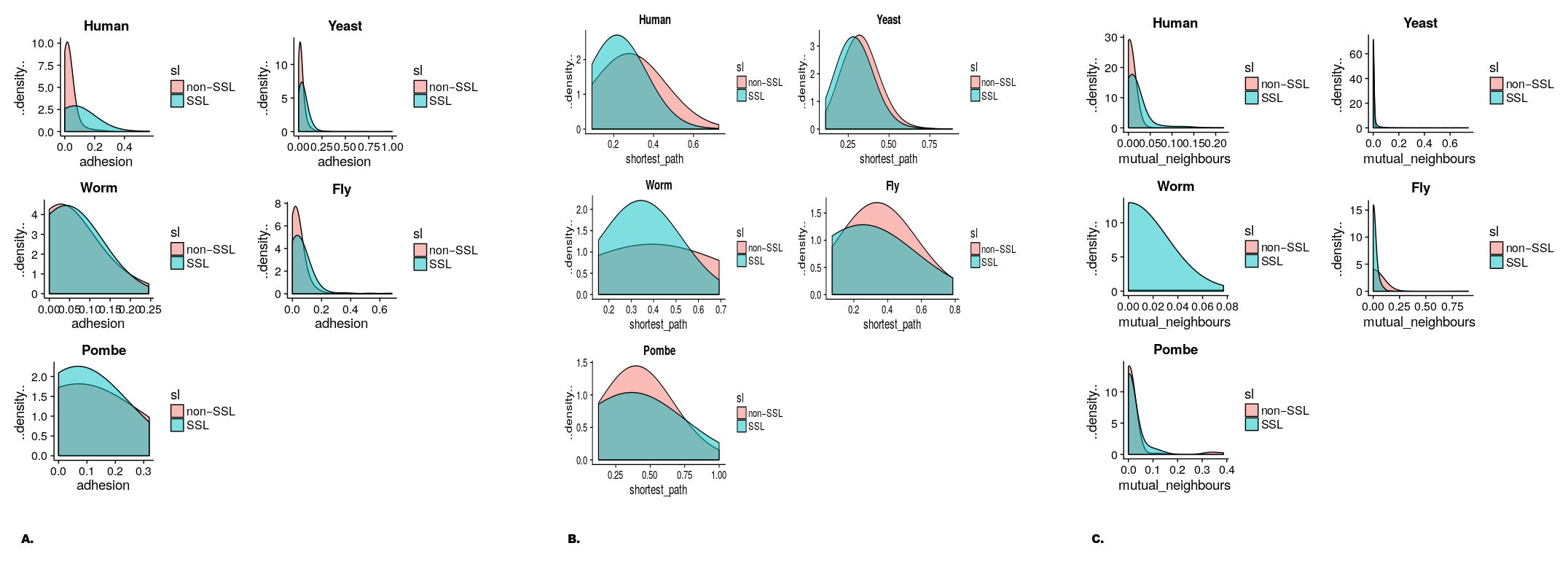

Supplement: S1 Fig — a. A distribution of normalised adhesion scores for each organism illustrate significant differences in SSL and non-SSL pairs across species. b. A normalised shortest path distribution shows a general trend for shorter shortest paths between H. sapiens SSL pairs though this difference is less pronounced in our model organisms. c. A distribution of normalised mutual neighbour counts suggests that SSL pairs often share more mutual neighbours than non-SSL pairs. (TIFF) [file pcbi.1006888.s001.tiff]

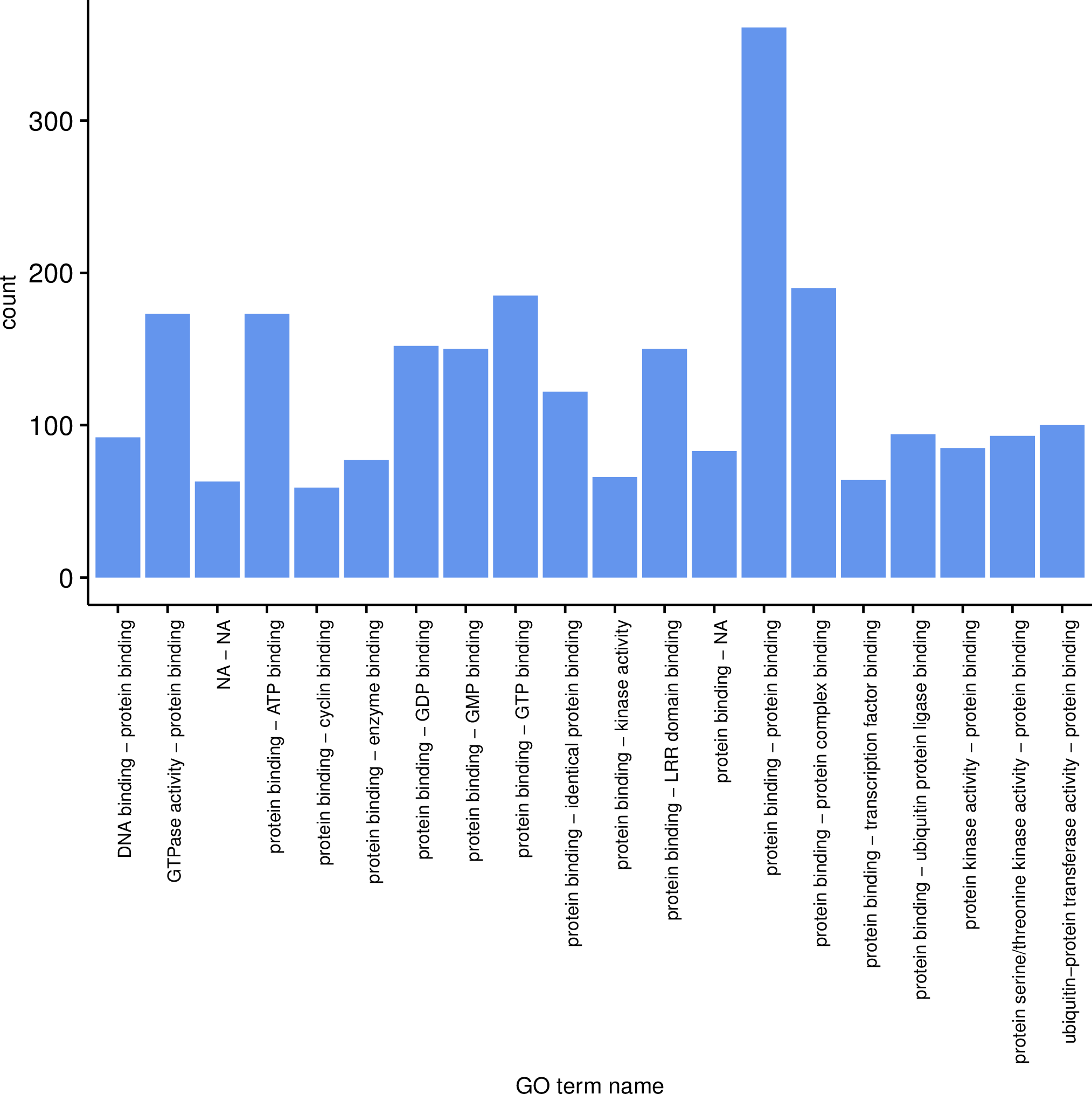

Supplement: S2 Fig — Count of most common associations between molecular function GO terms observed in SSL pairs. Individual feature GO associations extracted from full GO annotation lists for each SSL gene pair. (TIF) [file pcbi.1006888.s002.tif]

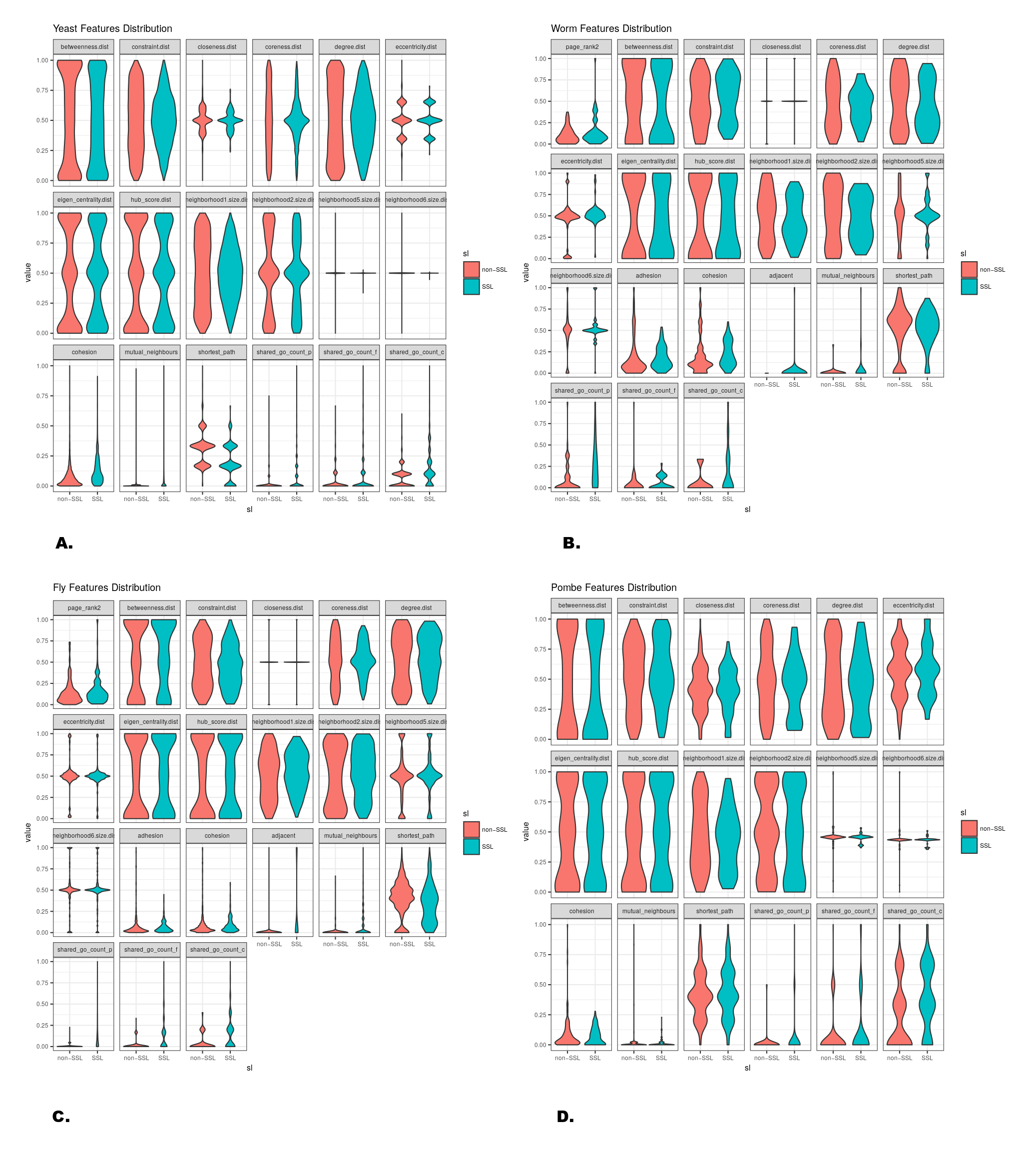

Supplement: S3 Fig — Violin plots illustrating feature value distributions for A, S. cerevisiae, B, C. elegans, C, D. melanogaster and D, S. pombe. (TIFF) [file pcbi.1006888.s003.tiff]

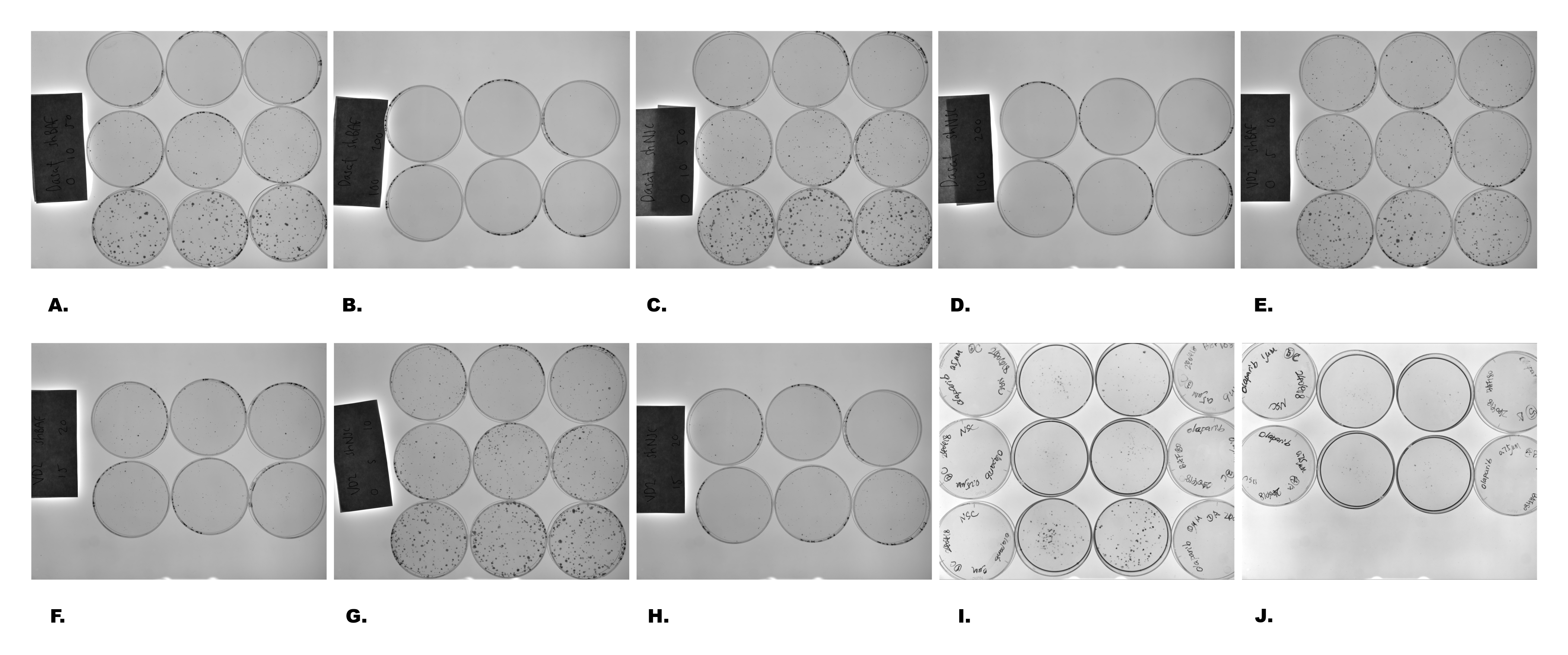

Supplement: S5 Fig — Survival assay plate images for ABL inhibitor Dasatanib (marked as Dasat) (A, B, C & D) and POLA inhibitor Erocalciferol (marked as VD2, an abbreviation of vitamin D2) experiments (E, F, G & H). BAF180 knock-out cell-line plate images for the PARP1 inhibitor Olaparib BAF180 are labeled with BAF and control plates marked with NSC on plate lids and the corresponding plate colonies are displayed adjacent to each lid (I & J). (TIFF) [file pcbi.1006888.s005.tiff]
