# Supplementary material for: Predicting synthetic lethal interactions using conserved patterns in protein interaction networks
Source: PLoS Comput Biol. 2019 Apr 17;15(4):e1006888. doi: 10.1371/journal.pcbi.1006888 (PMC6488098; doi:10.1371/journal.pcbi.1006888)
Supplement: S1 References — (DOCX) [file pcbi.1006888.s006.docx]

**Supplementary References**

1. Tangutoori S, Baldwin P, Sridhar S. PARP inhibitors: A new era of targeted therapy. Maturitas. 2015;81:5–9. doi:10.1016/j.maturitas.2015.01.015.

2. Jani JP, Finn RS, Campbell M, Coleman KG, Connell RD, Currier N, et al. Discovery and pharmacologic characterization of CP-724,714, a selective ErbB2 tyrosine kinase inhibitor. Cancer Res. 2007;67:9887–93.

3. Hall-Jackson CA, Eyers PA, Cohen P, Goedert M, Boyle FT, Hewitt N, et al. Paradoxical activation of Raf by a novel Raf inhibitor. Chem Biol. 1999;6:559–68.

4. Mizushina Y, Xu X, Murakami C, Okano T, Takemura M, Yoshida H, et al. Selective inhibition of mammalian DNA polymerase alpha by vitamin D2 and D3. J Pharmacol Sci. 2003;92:283–90. doi:10.1254/jphs.92.283.

5. Plimack ER, Lorusso PM, McCoon P, Tang W, Krebs AD, Curt G, et al. AZD1480: a phase I study of a novel JAK2 inhibitor in solid tumors. Oncologist. 2013;18:819–20. doi:10.1634/theoncologist.2013-0198.

6. Raju U, Riesterer O, Wang ZQ, Molkentine DP, Molkentine JM, Johnson FM, et al. Dasatinib, a multi-kinase inhibitor increased radiation sensitivity by interfering with nuclear localization of epidermal growth factor receptor and by blocking DNA repair pathways. Radiother Oncol. 2012;105:241–9. doi:10.1016/j.radonc.2012.08.010.

7. Rahmani M, Aust MM, Attkisson E, Williams DC, Ferreira-Gonzalez A, Grant S. Dual inhibition of Bcl-2 and Bcl-xL Strikingly Enhances PI3K inhibition-induced apoptosis in human myeloid leukemia cells through a GSK3- and bim-dependent mechanism. Cancer Res. 2013;73:1340–51.

8. Bastian M, Heymann S, Jacomy M. Gephi: An Open Source Software for Exploring and Manipulating Networks. Third Int AAAI Conf Weblogs Soc Media. 2009;:361–2. doi:10.1136/qshc.2004.010033.
