# Supplementary material for: Predicting synthetic lethal interactions using conserved patterns in protein interaction networks
Source: PLoS Comput Biol. 2019 Apr 17;15(4):e1006888. doi: 10.1371/journal.pcbi.1006888 (PMC6488098; doi:10.1371/journal.pcbi.1006888)
Supplement: S1 Table — Data is shown for A, H. sapiens, B, S. cerevisiae, C, C. elegans, D, D. melanogaster, and E, S. pombe. We observe that in humans SSL pairs share significantly more molecular function and cellular compartment GO terms while non-SSL pairs share significantly more biological process terms. A welch 2 sample t-test was used to measure significance for each annotation. 2.2e-16 was the smallest value available. (DOCX) [file pcbi.1006888.s007.docx]

| **GO Annotation type** | ***H. sapiens* shared GO terms** | **SSL** | **Non-SSL** | **Welch 2 sample t-test (p)** |
| --- | --- | --- | --- | --- |
| **Molecular function** | Mean | 1.195 | 0.733 | **<** **2.2e-16** |
|  | Stdev. | 0.903 | 0.682 |  |
| **Biological process** | Mean | 0.837 | 0.149 | **<** **2.2e-16** |
|  | Stdev. | 1.554 | 0.550 |  |
| **Cellular compartment** | Mean | 1.679 | 0.817 | **<** **2.2e-16** |
|  | Stdev. | 1.210 | 0.991 |  |

**Supplementary table 1A.**

| **GO Annotation type** | ***S. cerevisiae* shared GO terms** | **SSL** | **Non-SSL** | **Welch 2 sample t-test (p)** |
| --- | --- | --- | --- | --- |
| **Molecular function** | Mean | 0.486 | 0.258 | **< 2.2e-16** |
|  | Stdev. | 1.099 | 0.681 |  |
| **Biological process** | Mean | 0.760 | 0.136 | **< 2.2e-16** |
|  | Stdev. | 1.364 | 0.504 |  |
| **Cellular compartment** | Mean | 1.355 | 0.674 | **< 2.2e-16** |
|  | Stdev. | 1.369 | 0.825 |  |

**Supplementary table 1B.**

| **GO Annotation type** | ***C. elegans* shared GO terms** | **SSL** | **Non-SSL** | **Welch 2 sample t-test (p)** |
| --- | --- | --- | --- | --- |
| **Molecular function** | Mean | 0.423 | 0.229 | **< 2.2e-16** |
|  | Stdev. | 0.970 | 0.643 |  |
| **Biological process** | Mean | 2.368 | 0.683 | **2.642e-07** |
|  | Stdev. | 2.290 | 1.210 |  |
| **Cellular compartment** | Mean | 0.483 | 0.256 | **1.89e-08** |
|  | Stdev. | 0.772 | 0.510 |  |

**Supplementary table 1C.**

| **GO Annotation type** | ***D. melanogaster* shared GO terms** | **SSL** | **Non-SSL** | **Welch 2 sample t-test (p)** |
| --- | --- | --- | --- | --- |
| **Molecular function** | Mean | 0.554 | 0.099 | **< 2.2e-16** |
|  | Stdev. | 0.843 | 0.332 |  |
| **Biological process** | Mean | 1.842 | 0.098 | **< 2.2e-16** |
|  | Stdev. | 2.993 | 0.332 |  |
| **Cellular compartment** | Mean | 0.619 | 0.179 | **< 2.2e-16** |
|  | Stdev. | 0.829 | 0.428 |  |

**Supplementary table 1D.**

| **GO Annotation type** | ***S. pombe* shared GO terms** | **SSL** | **Non-SSL** | **Welch 2 sample t-test (p)** |
| --- | --- | --- | --- | --- |
| **Molecular function** | Mean | 0.252 | 0.107 | **0.2934** |
|  | Stdev. | 0.519 | 0.331 |  |
| **Biological process** | Mean | 0.0841 | 0.025 | **2.832e-09** |
|  | Stdev. | 0.312 | 0.173 |  |
| **Cellular compartment** | Mean | 1.16 | 0.623 | **< 2.2e-16** |
|  | Stdev. | 0.837 | 0.776 |  |

**Supplementary table 1E.**
