# Supplementary material for: Predicting synthetic lethal interactions using conserved patterns in protein interaction networks
Source: PLoS Comput Biol. 2019 Apr 17;15(4):e1006888. doi: 10.1371/journal.pcbi.1006888 (PMC6488098; doi:10.1371/journal.pcbi.1006888)
Supplement: S2 Table — This table contains a list of most important features for each species reported via the R caret libraries random forest classifier. Feature importance rankings were calculated by measuring the mean decrease in accuracy without each variable across all tree permutations in the random forest. (DOCX) [file pcbi.1006888.s008.docx]

|  | *H.*  *sapiens* | *S. cerevisiae* | *C.*  *elegans* | *D. melanogaster* | *S.*  *pombe* |
| --- | --- | --- | --- | --- | --- |
| Adhesion | ✔ |  |  |  |  |
| Adjacent |  |  |  | ✔ |  |
| Cohesion | ✔ | ✔ |  |  |  |
| Mutual neighbours |  | ✔ |  | ✔ |  |
| Shared GO Count – cellular compartment | ✔ | ✔ |  | ✔ | ✔ |
| Shared GO count – molecular function |  |  | ✔ |  |  |
| Shared GO Count – biological process | ✔ |  | ✔ | ✔ |  |
| Coreness | ✔ | ✔ | ✔ |  | ✔ |
| Neighborhood  size |  |  | ✔ |  | ✔ |
