# Supplementary material for: Predicting synthetic lethal interactions using conserved patterns in protein interaction networks
Source: PLoS Comput Biol. 2019 Apr 17;15(4):e1006888. doi: 10.1371/journal.pcbi.1006888 (PMC6488098; doi:10.1371/journal.pcbi.1006888)
Supplement: S3 Table — The best score for each species model is highlighted in green. Consensus model results are highlighted in blue. (DOCX) [file pcbi.1006888.s009.docx]

| **Model** | **Cross-species SDL classification performance (ROC AUC )** | |
| --- | --- | --- |
|  | *H.  sapiens* | *S.  cerevisiae* |
| *H. sapiens* | 0.782 | 0.754 |
| *S. cerevisiae* | 0.736 | 0.890 |
| Consensus | 0.805 | 0.918 |
