# Supplementary material for: Predicting synthetic lethal interactions using conserved patterns in protein interaction networks
Source: PLoS Comput Biol. 2019 Apr 17;15(4):e1006888. doi: 10.1371/journal.pcbi.1006888 (PMC6488098; doi:10.1371/journal.pcbi.1006888)
Supplement: S4 Table — (DOCX) [file pcbi.1006888.s010.docx]

| **Gene1** | **Gene2** | **Consensus score** |
| --- | --- | --- |
| SREBF1 | VHL | 0.810066584 |
| PTEN | SFN | 0.808599164 |
| RBX1 | VHL | 0.808448941 |
| PTEN | CHEK2 | 0.808017586 |
| UBE2D3 | VHL | 0.807876266 |
| BRAF | PTEN | 0.807264817 |
| FBXW7 | VHL | 0.806462054 |
| PTEN | CTNND1 | 0.806458647 |
| GSK3B | VHL | 0.805455056 |
| APC | AURKB | 0.805347415 |
| APC | CTNND1 | 0.805335746 |
| PIN1 | PTEN | 0.805318639 |
| MAP3K1 | PTEN | 0.805219709 |
| CDKN1B | PTEN | 0.804986161 |
| ORC1 | PTEN | 0.80472497 |
| ARRB2 | PTEN | 0.804692535 |
| SKP2 | PTEN | 0.80468009 |
| BUB1B | PTEN | 0.803596615 |
| VHL | NOTCH1 | 0.803412785 |
