# Supplementary material for: Predicting synthetic lethal interactions using conserved patterns in protein interaction networks
Source: PLoS Comput Biol. 2019 Apr 17;15(4):e1006888. doi: 10.1371/journal.pcbi.1006888 (PMC6488098; doi:10.1371/journal.pcbi.1006888)
Supplement: S5 Table — We chose a group of genes with selective inhibitors that were predicted to share a synthetic lethal interaction with BAF180 (PBRM1) for validation. We performed clonogenic survival assays for each inhibitor using U2OS cell lines (shControl + mCherry/NLS and shBAF180 + GFP/NLS). (DOCX) [file pcbi.1006888.s011.docx]

| **Drug** | **Target** | **Citation** |
| --- | --- | --- |
| Olaparib | PARP1 | [1] |
| CP-724714 | ERBB2/HER2 | [2] |
| ZM 336372 | RAF1 | [3] |
| Ergocalciferol (Vitamin D2) | POLA1 | [4] |
| AZD1480 | JAK2 | [5] |
| Dasatinib | ABL1 | [6] |
| CHIR-98014 | GSK3B | [7] |
