# Supplementary material for: Predicting synthetic lethal interactions using conserved patterns in protein interaction networks
Source: PLoS Comput Biol. 2019 Apr 17;15(4):e1006888. doi: 10.1371/journal.pcbi.1006888 (PMC6488098; doi:10.1371/journal.pcbi.1006888)
Supplement: S6 Table — Number of SSL pairs and SDL pairs sourced for each organism from BioGRID after filtering for distinct pairs that inlcude genes present in the protein interaction network. The SSL pair data for S. cerevisiae were filtered to include only interactions cited in 3 or more papers. SSL pair data for S. pombe were filtered to include only interactions recorded in 2 or more papers. (DOCX) [file pcbi.1006888.s012.docx]

| **Organism** | **Protein interactions** | **SSL / Negative GI count** | **SDL count** |
| --- | --- | --- | --- |
| *H. sapiens* | 60,278 | 411 | 259 |
| *S. cerevisiae* | 82,480 | 17,568 (of 395,199) | 2,389 |
| *C. elegans* | 36,332 | 1,237 | 0 |
| *D. melanogaster* | 34,324 | 348 | 0 |
| *S. pombe* | 47,492 | 3836 (of 35,391) | 0 |
