# Supplementary material for: Predicting synthetic lethal interactions using conserved patterns in protein interaction networks
Source: PLoS Comput Biol. 2019 Apr 17;15(4):e1006888. doi: 10.1371/journal.pcbi.1006888 (PMC6488098; doi:10.1371/journal.pcbi.1006888)
Supplement: S7 Table — SLant also treats node-wise features differently by providing an averaged difference between node pairs as well as the individual values per gene node. (DOCX) [file pcbi.1006888.s013.docx]

| **Feature** | **SLant** | **SINaTRA** |
| --- | --- | --- |
| Betweenness | x | x |
| Constraint | x |  |
| Closeness | x | x |
| Coreness | x |  |
| Degree | x | x |
| Eccentricity | x | x |
| Eigen centrality | x | x |
| Hub score | x |  |
| Neighbourhood n size | x | x |
| PageRank |  | x |
| Adhesion | x |  |
| Cohesion | x |  |
| Communicability |  | x |
| Current-flow betweenness centrailty |  | x |
| Adjacent | x |  |
| Mutual neighbours | x | x |
| Mutual non-neighbours |  | x |
| Shortest path | x |  |
| Inversed shortest path |  | x |
| Between community | x |  |
| Cross community | x |  |
| Shared GO count – Biological process | x |  |
| Shared GO count – Molecular function | x |  |
| Shared GO count – Cellular compartment | x |  |
