# Supplementary material for: Predicting synthetic lethal interactions using conserved patterns in protein interaction networks
Source: PLoS Comput Biol. 2019 Apr 17;15(4):e1006888. doi: 10.1371/journal.pcbi.1006888 (PMC6488098; doi:10.1371/journal.pcbi.1006888)
Supplement: S8 Table — SLant data were generated in house, SINaTRA scores were extracted from Jakunski et al., 2015 publication. (DOCX) [file pcbi.1006888.s014.docx]

|  | *SLant  (BioGRID 3.2.104)* | *SINaTRA (Published, 2015 ) (BioGRID 3.2.104)* |
| --- | --- | --- |
| *S. cerevisiae* | *0.98 (Cross validation)* | *0.92 (Cross validation)* |
| *S. pombe* | *0.98(Cross validation)* | *0.93 (Cross validation)* |
| *S. cerevisiae to S. pombe* | *0.88* | *0.86* |
| *S. pombe to S. cerevisiae* | *0.77* | *0.74* |
