# Supplementary material for: Predicting synthetic lethal interactions using conserved patterns in protein interaction networks
Source: PLoS Comput Biol. 2019 Apr 17;15(4):e1006888. doi: 10.1371/journal.pcbi.1006888 (PMC6488098; doi:10.1371/journal.pcbi.1006888)
Supplement: S9 Table — AUC ROC scores from the SLant feature set versus the SINaTRA feature set using the full current training sets and the pairwise non-bias data sets. (DOCX) [file pcbi.1006888.s015.docx]

|  | *Slant* | *SLant (pairwise unbiased data)* | *SINaTRA features  (BioGrid 3.4.156)* | *SINaTRA features (pairwise unbiased data)* |
| --- | --- | --- | --- | --- |
| *S. cerevisiae* | *0.907 (Consensus)* | *0.803 (Consensus)* | *0.893* | *0.726* |
| *S. pombe* | *0.920 (Consensus)* | *0.835 (Consensus)* | *0.912* | *0.572* |
| *S. cerevisiae to S. pombe* | *0.717* | *0.701* | *0.680* | *0.652* |
| *S. pombe to S. cerevisiae* | *0.607* | *0.663* | *0.609* | *0.534* |
