# Supplementary material for: Predicting synthetic lethal interactions using conserved patterns in protein interaction networks
Source: PLoS Comput Biol. 2019 Apr 17;15(4):e1006888. doi: 10.1371/journal.pcbi.1006888 (PMC6488098; doi:10.1371/journal.pcbi.1006888)
Supplement: S10 Table — A comparison of human SSL classification using the SLant consensus set versus the SINaTRA feature set using current data. (DOCX) [file pcbi.1006888.s016.docx]

|  | *Model* | *All training data* | *Non-bias data* |
| --- | --- | --- | --- |
| *Human classification* | *SLant Consensus* | *0.985* | *0.789* |
|  | *Sinatra features* | *0.966* | *0.451* |
